# Multifunctional Carbon Nanodots: Enhanced Near‐Infrared Photosensitizing, Photothermal Activity, and Body Clearance

**DOI:** 10.1002/smsc.202100082

**Published:** 2021-12-05

**Authors:** Ding-Kun Ji, Hayet Dali, Shi Guo, Sowmya Malaganahally, Julien Vollaire, Véronique Josserand, Hélène Dumortier, Cécilia Ménard-Moyon, Alberto Bianco

**Affiliations:** ^1^ CNRS, Immunology, Immunopathology and Therapeutic Chemistry, UPR 3572 University of Strasbourg, ISIS Strasbourg 67000 France; ^2^ Institut pour l'Avancée des Biosciences, INSERM U1209 CNRS UMR-5309, Université Grenoble Alpes Grenoble 38000 France; ^3^ Present address: Institute of Molecular Medicine (IMM) Renji Hospital School of Medicine Shanghai Jiao Tong University Shanghai 200240 China

**Keywords:** carbon dots, carbon materials, covalent modifications, fluorescence imaging, nanoprobes, phototherapy

## Abstract

The emergence of quasispherical carbon nanodots (CNDs) has broadened the family of carbon‐based materials and their applications in many fields. However, the rational design of near‐infrared (NIR) CNDs and their specific applications in theranostic biomedicine are still challenging. Herein, the design and characterization of multifunctional CNDs (MCNDs) as NIR laser‐triggered nanoagents are reported. These MCNDs are constructed by covalent functionalization of CNDs with folic acid and a hydrophobic photosensitizer, chlorin e6 (Ce6), based on a double amidation approach. The resulting MCNDs show high colloidal stability in different physiological environments. They are endowed with NIR fluorescence emission, an enhanced NIR photosensitizing, and a high photothermal activity. The in vitro test indicates that MCNDs own an efficient targeting capacity to penetrate into cancer cells, leading to cell killing at a low dose under NIR laser irradiation. Finally, MCNDs display a long blood circulation time in mice and effective body clearance through a dual hepatobiliary and renal elimination pathway. These features are encouraging for their use as NIR laser‐triggered nanoagents for personalized medicine. This work not only paves a new way for the multifunctionalization of CNDs, simply by integrating other functional components, but also broadens their versatile applications in targeted cancer theranostic nanomedicine.

## Graphical abstract

A flexible method of double covalent functionalization of carbon nanodots (CNDs) is developed based on a double amidation approach. The resultant multifunctional CNDs own high hydrophobic Ce6 loading, near‐infrared (NIR) photosensitizing, photothermal activity, effective targeted cancer phototherapy, and body clearance. Herein, an efficient strategy for covalent multifunctionalization of CNDs to design the second generation of “all‐in‐one” theranostic reagents is provided.

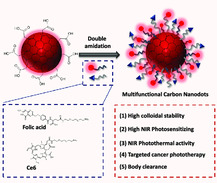

## 1 Introduction

Photodynamic therapy (PDT) is an emerging cancer therapy method which is considered a minimally invasive treatment for various premalignant and neoplastic diseases.^[^ ^1^ ^]^ Compared with other traditional treatments (surgery, radiotherapy, and chemotherapy), PDT exhibits several unique advantages, including a negligible drug resistance, reduced side effects, and less damage to marginal tissues.^[^ ^2^ ^]^ Photosensitizers (PSs) play an important role in PDT by producing cytotoxic reactive oxygen species (ROS) when irradiated with an appropriate light, resulting in the elimination of tumor cells. To date, various organic PSs, such as porphyrin, phthalocyanines, and bacteriochlorin derivatives, possess simultaneous cancer imaging and therapeutic capabilities, and some of them have been approved for clinical use for cancer treatment.^[^ ^3^, ^4^ ^]^ However, the clinical applications of organic PSs are often constrained by drawbacks associated with poor water solubility, a low targeting capacity, and an inability to absorb in the near‐infrared (NIR) region above 700 nm, where the biological barriers (e.g., skin) are most transparent.^[^ ^5^, ^6^ ^]^

To overcome this issue, nanoparticle‐based carriers can be used, as they can be loaded with a large number of hydrophobic drugs, including PSs, and deliver them to tumors through either a passive or an active targeting approach.^[^ ^7^, ^8^ ^]^ Despite extensive research, the current nanotheranostic agents share some common drawbacks. For instance, the PS molecules may be released from the nanoparticles before reaching the lesion, leading to collateral damage to normal tissues. Moreover, some nanocarriers may be toxic and their clearance from the body might be slow and inefficient.^[^ ^9^ ^]^ To this end, a water‐dispersible nanovehicle with a high loading capacity of NIR‐responsive hydrophobic PSs, negligible toxicity, and the possibility to modulate its surface properties through functionalization would be ideal for phototherapy in clinic.

In this context, water‐dispersible carbon nanodots (CNDs) have recently received considerable attention in biosensing, bioimaging, drug delivery, and phototherapy, by virtue of their excellent optical properties, low price of production, high water dispersibility, low toxicity, and high biocompatibility.^[^ ^10^ ^]^ In particular, benefiting from abundant reactive groups on their surface, CNDs can act as promising carriers by covalent conjugation or coassembly with functional dyes, drugs, or proteins, resulting in a range of novel multifunctional nanomedicines.^[^ ^11^ ^]^ As an example, we previously demonstrated that a targeted intracellular production of ROS could be successfully achieved in tumor cells using folic acid (FA)‐modified photoactive CNDs,^[^ ^12^ ^]^ while the covalent functionalization of gadolinium to CND surface allowed multimodal bioimaging.^[^ ^13^ ^]^ Chlorin e6 (Ce6) is a second‐generation PS with a high efficacy and low dark toxicity, but its extremely poor water solubility and instability hamper its clinical use.^[^ ^14^ ^]^ The combined irradiation of both CNDs and Ce6 could enhance the ROS generation, thus boosting the whole PDT efficiency. Indeed, CNDs have been successfully applied for the delivery of Ce6 via a noncovalent or covalent method for in vivo fluorescence imaging‐guided PDT or/and photothermal therapy (PTT).^[^ ^15^, ^16^, ^17^ ^]^ However, due to their ultrasmall size, the rational design of NIR‐triggered CND‐based nanostructures with Ce6 and other functional groups is still challenging. To further increase the functionality of CNDs, developing multifunctionalization strategies is highly required.

We herein present a flexible method for constructing multifunctional CNDs (MCNDs) by covalent double functionalization with FA and Ce6. Water‐dispersible CNDs were synthesized via hydrothermal treatment of a conjugated polymer. Then, FA, which is specifically recognized by the folate receptor (FR) overexpressed in many diseased cells, and Ce6 were both covalently attached to the surface of CNDs by a double amidation strategy. The MCNDs showed a good dispersibility in different physiological environments, eliminating the need of an organic solvent, polyethylene glycol, a surfactant, or oil as cosolubilizer. These dots emitting in the NIR region displayed enhanced NIR photosensitizing properties and good photothermal conversion efficiency. In vitro tests on HeLa cells indicated that MCNDs easily penetrated the cells, further killing them already at a low dose (10 μg mL^−1^), once irradiated by an NIR laser. They displayed limited intrinsic cytotoxicity and proinflammatory properties, a long blood circulation time, and an effective hepatobiliary and renal clearance. Overall, this study demonstrates the feasibility of using CNDs for efficient and safe targeted delivery of hydrophobic drugs to cancer cells. Taken together, these features of MCNDs are encouraging for their use as NIR laser‐triggered nanoagents for personalized medicine.

## 2 Results and Discussion

### 2.1 Preparation of MCNDs

**Scheme 1:**
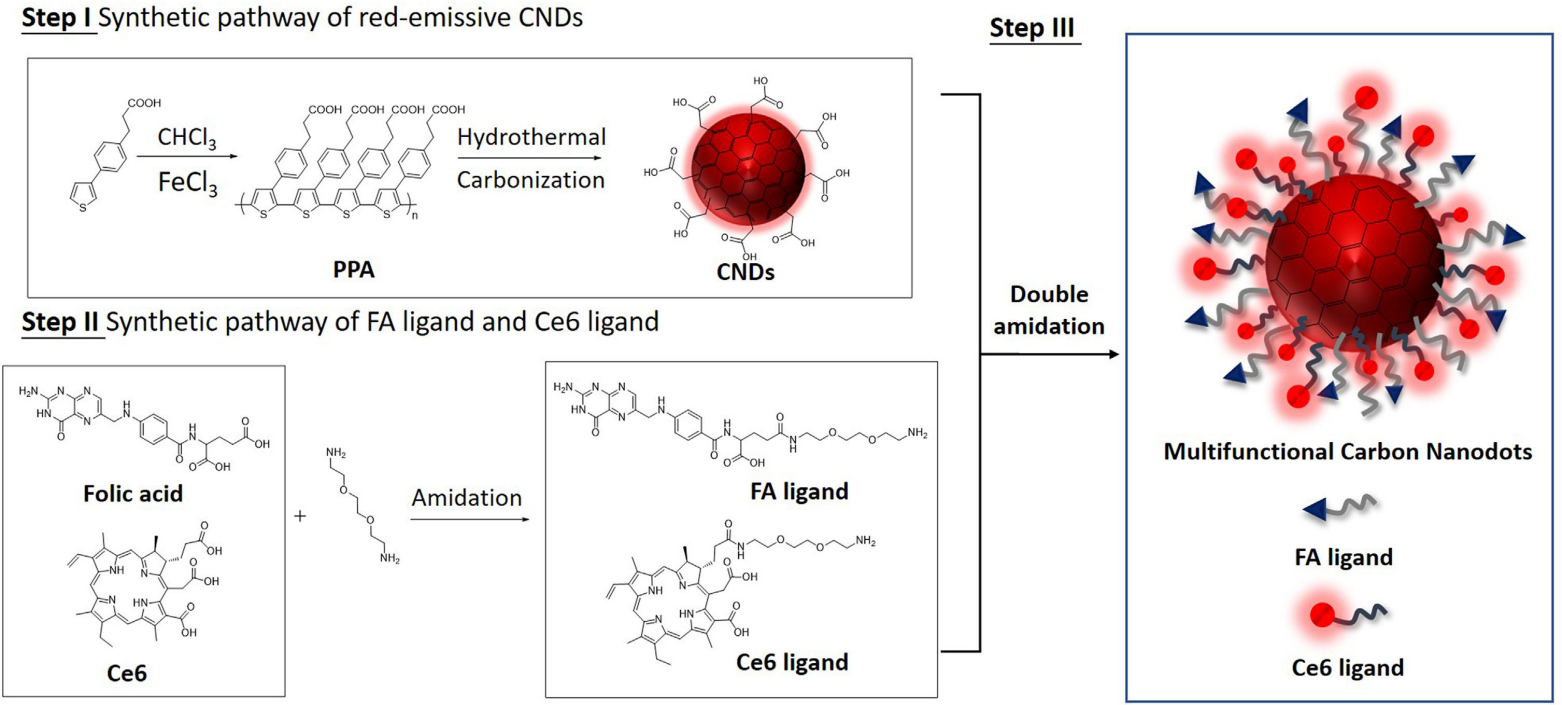
Synthesis of CNDs and subsequent functionalization with FA and Ce6. Step I, synthesis of CNDs; Step II, synthesis of FA and Ce6 ligands; and Step III, double covalent modification of CNDs with FA and Ce6.

Based on our previous work,^[^ ^12^ ^]^ carboxyl‐rich CNDs were first synthesized by a hydrothermal method using a conjugated polymer, polythiophene phenylpropionic acid (PPA), as the carbon source (**Scheme** **1**, Step I). A short amine‐terminated triethylene glycol (TEG) chain was chosen as a linker to connect CNDs with FA and Ce6. For this purpose, FA−TEG−NH_2_ and Ce6−TEG−NH_2_ ligands were designed and prepared according to **Scheme** **1** (Step II). A previous paper reported that only one of the carboxylic groups of Ce6 can be selectively functionalized by TEG.^[^ ^18^ ^]^ As suggested in this work, the COOH attached to Ce6 through the longer alkyl chain is the most reactive. We have optimized our synthesis to derivatize only one COOH. Our NMR and mass spectrometry (MS) data are consistent with monofunctionalization; however, we cannot exclude that the other two COOH also react with TEG, thus leading to a mixture of monofunctionalized products. The modification does not affect the properties of Ce6. For simplicity, we have represented the TEG attached only to the carboxylic function on the longest methylene chain. The introduction of a short TEG chain could enhance FA and Ce6 water solubility and avoid a potential quenching effect between Ce6 and CNDs. It also offered the advantage of minimizing the adsorption of unreacted precursors, facilitating their removal, compared with longer ethylene glycol chains.^[^ ^19^ ^]^ FA ligand was first conjugated to the COOH groups of CNDs. Considering the second coupling reaction with Ce6 ligand, the amount of FA ligand was well controlled not to saturate all the COOH groups of CNDs. FA ligand could not only endow CNDs with targeting ability, but also enhance their colloidal stability. Finally, Ce6 ligand was covalently attached to the surface of the monofunctionalized CNDs (FCNDs) by amidation to obtain MCNDs (Scheme 1, Step III).

### 2.2 Characterization of the MCNDs

**Figure 1:**
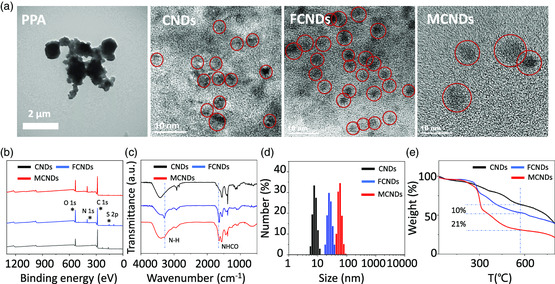
Characterization of the nanoparticles. a) TEM images of PPA and high‐resolution TEM images of CNDs, FCNDs, and MCNDs; b) XPS, c) FTIR, d) DLS, and e) TGA of CNDs, FCNDs, and MCNDs.

The morphology and structure of PPA, CNDs, FCNDs, and MCNDs was first characterized by transmission electron microscopy (TEM). As shown in **Figure** **1**a, the as‐prepared PPA polymer appeared as large aggregates. After the hydrothermal process, the resulting CNDs had a diameter below 5 nm. After conjugation of FA and Ce6, FCNDs and MCNDs displayed a bigger size and a smoother edge than CNDs. The elemental composition of CNDs, FCNDs, and MCNDs was obtained by X‐ray photoelectron spectroscopy (XPS). As shown in Figure 1b, the spectrum of CNDs contained three main peaks corresponding to O 1*s* (532.8 eV), S 2*p* (164.1 eV), and C 1*s* (284.1 eV), suggesting that the as‐prepared CNDs are sulfur‐doped nanoparticles (Figure S1, Supporting Information). The C 1*s* peak was deconvoluted into four peaks: graphitic (C=C) and aliphatic (C—C), C—O/C—S, C=O, and COOH (Figure S2, Supporting Information). Both FCNDs and MCNDs showed the appearance of the N 1*s* peak at 400.1 eV and a peak attributed to N—C=O at 287.8 eV (Figure S1, S2, Supporting Information). The content of N increased from 7.1% to 10.8% after the second reaction, confirming the amidation of CNDs with FA and Ce6 ligands. FTIR allowed to evidence the presence of the N−H stretching and amide I stretching at 3296 and 1640 cm^−1^, respectively (Figure 1c). Thus, these results collectively confirm that FA and Ce6 were successfully covalently grafted onto the surface of CNDs. Their hydrodynamic size distribution was then measured using dynamic light scattering (DLS). All three nanoparticles exhibited a homogenous and narrow size distribution, corresponding to 5−12 nm for CNDs, 10−30 nm for FCNDs, and 40−90 nm for MCNDs (Figure 1d). Their average hydrodynamic size was 7.5, 20, and 50 nm, respectively. These values do not represent the precise size of CNDs. Indeed, the size distribution of nanoparticles assessed by DLS is usually larger than that obtained by other techniques like TEM, as DLS measures the hydrodynamic size of the core and takes into account also the surrounding solvation molecules. In addition, DLS is more accurate for soft materials, such as polymers or proteins, while the size of hard materials may be overestimated.^[^ ^20^, ^21^ ^]^ To calculate the accurate amount of FA and Ce6 ligands on the surface of MCNDs, thermogravimetric analysis (TGA) of CNDs, FCNDs, and the final conjugates was performed under inert atmosphere. An increasing weight loss at 600 °C was observed from CNDs to FCNDs (10%) and MCNDs (31%), further confirming the efficiency of double functionalization (Figure 1e). The precise amount of FA and Ce6 was calculated as 227 and 352 μmol g^−1^, respectively. Taken together, all these data suggest the successful preparation of both FA and Ce6‐modified CNDs.

### 2.3 Optical Properties of MCNDs

**Figure 2:**
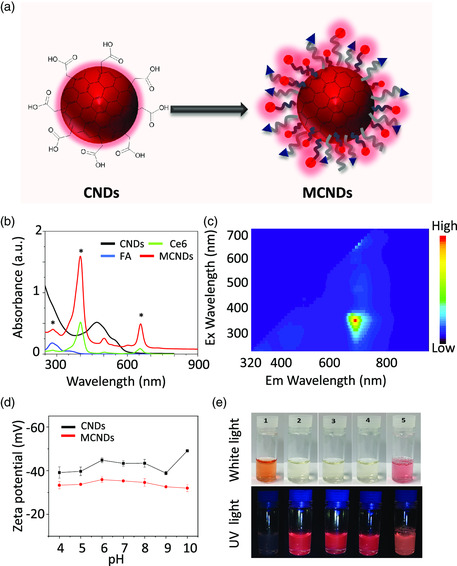
a) Schematic of CNDs and MCNDs. b) UV−vis spectra of FA, Ce6, CNDs, and MCNDs (50 μg mL^−1^ in PBS, pH = 7.4). c) Fluorescence of MCNDs (320−900 nm) for excitation wavelengths from 300 to 700 nm. d) Zeta potential of CNDs and MCNDs in PBS buffer at different pH (4−10). e) Digital images of CNDs in PBS (1) and MCNDs (50 μg mL^−1^) in water (2), PBS (3), saline (4), and cell culture medium (5).

CNDs and MCNDs (**Figure** **2**a) were characterized by UV−vis−NIR and fluorescence spectroscopy. CNDs displayed UV absorption from 260 to 650 nm (Figure 2b). After being double covalently functionalized, the absorbance of as‐prepared MCNDs red‐shifts to 900 nm and displays additional peaks of Ce6 at 408 and 675 nm and FA at 282 nm. In particular, MCNDs displayed high absorption in the NIR region that could predict their potential capacity for NIR laser‐triggered PDT and/or PTT. The fluorescence emission spectrum of MCNDs under different excitation wavelengths was also measured. As shown in Figure 2c, MCNDs are characterized by a broad fluorescence emission spectrum from 400 to 800 nm, arising from the combination of both CNDs and Ce6 contributions (Figure S3, Supporting Information). The fluorescence quantum yields of CNDs, Ce6, and MCNDs were measured using a spectrometer attached to an integrating sphere and corresponded to 0.23%, 1.75%, and 4.32%, respectively. The fluorescence quantum yield of MCNDs is around 20 times higher than CNDs and 2.5 times higher than Ce6. In addition, the maximum emission peak of MCNDs at ≈670 nm is similar to that of free Ce6, which could be beneficial for NIR fluorescence imaging‐guiding therapy.

### 2.4 Dispersibility and Stability of MCNDs

A good stability and dispersibility of PSs in aqueous solutions are essential for efficient phototherapy. Unfortunately, although Ce6 is the most widely used second‐generation PS, its poor solubility in aqueous media has highly limited its clinical use in phototherapy. Thus, many efforts are focused on improving its water solubility by the conjugation of hydrophilic moieties, including dendrimers,^[^ ^22^ ^]^ polymers,^[^ ^23^ ^]^ or proteins.^[^ ^24^ ^]^ By exploiting the high water dispersibility of CNDs, we could significantly improve the aqueous solubility of Ce6. The stability of MCNDs and CNDs was scrutinized by measuring the zeta potential at different pH. Both nanoparticles exhibit a negative zeta potential at pH = 7, −34 ± 1.4 mV and −42 ± 3.7 mV for MCNDs and CNDs, respectively (Figure 2d). The lower negative value for MCNDs compared with CNDs was due to the attachment of FA and Ce6. Although original CNDs showed good water dispersibility, some aggregation was however observed in a pH range of 3−10 over a period of 24 h, probably due to strong H‐bonding interactions (Figure S4, Supporting Information).^[^ ^12^ ^]^ We also found that the zeta potential of CNDs becomes more negative at pH > 9.0, because the network of hydrogen bonds between CNDs likely starts to disintegrate in more basic conditions, leading to the formation of a higher number of isolated nanodots.^[^ ^25^ ^]^ In contrast, we did not observe any aggregation phenomenon in MCND samples in a broad pH range of 3−12 (Figure S5, Supporting Information), even for high concentration of MCNDs (up to 100 μg mL^−1^). These results are indicative of the superior colloidal stability of MCNDs. The covalent modifications with the TEG linkers could highly improve the stability of the nanoparticles at different pH. Furthermore, MCNDs were easily dispersed in different physiological solutions, including water, PBS, saline, and cell culture medium (Figure 2e). No precipitates were observed after storing the dispersions at room temperature over weeks (up to 1 mg mL^−1^), whereas Ce6 is insoluble in PBS at high concentrations (Figure S6, Supporting Information). MCNDs also displayed a stable and bright NIR fluorescence emission in physiological solution under UV irradiation (365 nm).

### 2.5 In Vitro Photothermal and Photodynamic Effect of MCNDs

**Figure 3:**
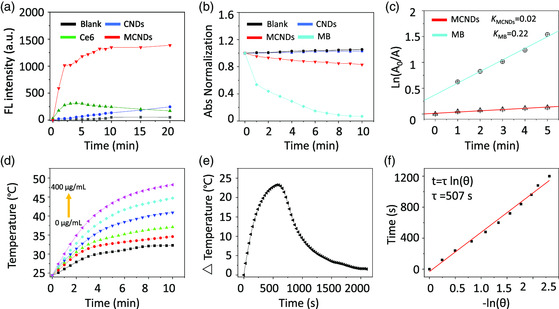
The PDT and PTT properties of MCNDs. a) The changes of fluorescence intensity at the characteristic emission peak of rhodamine 123 (585 nm) over 20 min laser irradiation at 660 nm. The concentration of MCNDs, Ce6, and DHR 123 was 25 μg mL^−1^, 5 μg mL^−1^, and 20 nM, respectively. b) Normalized absorbance of ABDA at 400 nm in the presence of CNDs, MCNDs, and MB under laser irradiation at 660 nm for a period of 10 min. c) Decay curves of the ABDA absorption at 400 nm as a function of irradiation time. d) Time‐dependent photothermal curves for increasing concentrations of MCND dispersion (0, 0.05, 0.1, 0.2, 0.3, 0.4 mg mL^−1^) under 808 nm laser irradiation (2 W cm^−2^). e) Evolution of the photothermal effect of MCND dispersion (0.4 mg mL^−1^) exposed to 808 nm laser irradiation (2 W cm^−2^) for 10 min. f) Plot of cooling time versus negative natural logarithm of the temperature driving force. The time constant *τ* was calculated and corresponds to 507 s.

Considering their high NIR absorption, the photodynamic and photothermal activities of MCNDs were evaluated. To monitor the ROS generation capability of MCNDs, dihydrorhodamine 123 (DHR 123) was used as a probe, as the reaction with ROS leads to the formation of rhodamine 123 with a high red‐emissive fluorescence. An aqueous dispersion containing MCNDs and DHR 123 showed a rapid increase in the fluorescence intensity at 585 nm upon exposure to 660 nm laser (0.1 W cm^−2^), which was around 5.5 times more than that generated by the same concentration of Ce6 in water (**Figure** **3**a and S7, Supporting Information). Furthermore, we observed that the ROS production of MCNDs was nine times higher than that generated by the same concentration of CNDs. As control, exposing DHR 123 solution alone to 660 nm laser irradiation did not change its fluorescence, thus indicating the fast and efficient ROS generation by MCNDs. The enhanced ROS generation benefits from the conjugation of Ce6 to CNDs that prevents aggregation and improves the solubility of the PS in aqueous solution. We further determined the singlet‐oxygen yield using 9,10‐anthracenediyl‐bis(methylene) dimalonic acid (ABDA) as probe and methylene blue (MB) as standard reference (MB singlet oxygen *η* = 0.52 in water).^[^ ^26^ ^]^ The underlying operational principle for ABDA involves the disappearance of its main absorption peak at 400 nm after reaction with ^1^O_2_.^[^ ^27^ ^]^ The fast degradation of ABDA in the presence of MCNDs under 660 nm laser irradiation compared with pure ABDA and CNDs (Figure 3b and S8, Supporting Information) further confirms the efficient ROS generation. Based on the decay curves of the ABDA absorption (Figure 3c), ^1^O_2_ yield of MCNDs was calculated to be 17.3%, according to a comparative method,^[^ ^26^, ^28^, ^29^ ^]^ which is quite close to the clinically used PS Photofrin (Φ = 0.28).^[^ ^30^ ^]^

The photothermal conversion efficiency of MCNDs was also evaluated. For this purpose, MCND dispersions with elevated concentrations (0.05, 0.1, 0.2, 0.3, 0.4 mg mL^−1^) were exposed to an 808 nm NIR laser (2 W cm^−2^) for 10 min and the temperature was measured by an IR thermal camera (Figure 3d). A strong concentration‐dependent photothermal effect was observed with a temperature increase of 23 °C for the highest concentration. Comparatively, as a control, the temperature of pure water (without MCNDs) only increased by less than 6 °C (data not shown). These results indicated that MCNDs can efficiently convert laser energy into heat. To evaluate the photothermal conversion efficiency of MCNDs, we recorded the temperature change of the solution at 0.4 mg mL^−1^ as a function of time under continuous irradiation at 808 nm (2 W cm^−2^) until the solution reached a steady‐state temperature (Figure 3e,f). According to previous reported methods,^[^ ^31^, ^32^, ^33^ ^]^ the photothermal conversion efficiency of MCNDs was calculated to be 25.9%.

### 2.6 Impact of MCNDs on Macrophages

**Figure 4:**
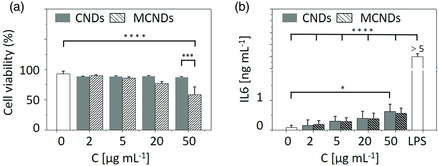
a) Cell viability and b) IL‐6 production upon incubation of RAW 264.7 murine macrophages with increasing concentrations of CNDs and MCNDs (0−50 μg mL^−1^) or LPS (positive control) for 24 h. Data are the mean ± sd (standard deviation) of three independent experiments (two‐way ANOVA with Bonferroni t‐test: *p < 0.05, ***p < 0.001, ****p < 0.0001).

The assessment of the potential cellular toxicity represents an important aspect toward the development of new types of PSs for phototherapy. The first step in our biological investigations was to determine the impact of CNDs and MCNDs on cells that most often are at the forefront upon administration of nanomaterials, that is, macrophages. For this purpose, RAW 264.7 murine macrophages were treated with increasing concentrations of CNDs and MCNDs (2, 5, 20, 50 μg mL^−1^) in the dark and cell viability was assessed by flow cytometry after 24 h of incubation. CNDs had no impact even at a high concentration of 50 μg mL^−1^ (88 ± 1% viability vs 93 ± 5% in the untreated control), while a dose‐dependent decrease was observed with MCNDs (59 ± 15% at 50 μg mL^−1^) (**Figure** **4**a). Nevertheless, at concentrations below 50 μg mL^−1^, the cell viability was above 75% (81% of the untreated control), indicating a weak intrinsic cytotoxicity. We also determined whether CNDs and MCNDs could induce a proinflammatory response in macrophages by measuring the release of cytokines, that is, IL‐6, IL‐1β, and TNF‐α, by ELISA. As shown in Figure 4b, we observed a dose‐dependent production of IL‐6 upon incubation with both types of CNDs; however, secreted amounts remained definitely lower (maximum 0.6 ± 0.25 ng mL^−1^) than what we measured when macrophages were stimulated with lipopolysaccharides (LPS, saturated signal, >5 ng mL^−1^). Similar profiles were obtained for TNF‐α and IL1‐β (data not shown). Overall, CNDs and MCNDs displayed limited intrinsic cytotoxicity and inflammatory properties.

### 2.7 Macrophage Uptake and Fluorescence Imaging of MCNDs

**Figure 5:**
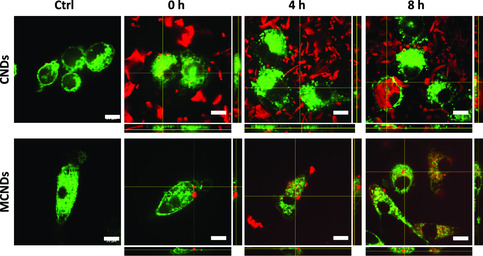
Fluorescence images of RAW 264.7 cells incubated with 100 μg mL^−1^ of CNDs and MCNDs for 0, 4, and 8 h. In green, membranes stained with CellMask (excitation channel: 488 nm, emission: 500−550 nm); in red, CNDs and MCNDs (excitation channel: 405 nm, emission: 665−715 nm). The scale bar is 20 μm.

Besides a good biocompatibility, the capability to reach the intracellular compartments is another key characteristic for promising PSs. In this regard, we studied the uptake of CNDs and MCNDs at the concentration of 100 μg mL^−1^ in RAW 264.7 macrophages for different incubation times (0, 4, and 8 h) (**Figure** **5**). CNDs and MCNDs were easily visualized by confocal microscopy in the NIR channel (665−715 nm) due to the fluorescence of both Ce6 and CNDs. The CellMask green dye (Thermo Fisher) was used for cell membrane staining. We observed that CNDs were highly aggregated outside the cells. Although macrophages own a strong phagocytic activity, almost no CNDs were internalized inside the cells, while the uptake of MCNDs started during the first 4 h and increased over time up to 8 h. We could clearly observe the cytoplasm full of red dots at 8 h at the tested concentration, proving the efficient internalization of MCNDs. Indeed, the double covalent modification could effectively inhibit the aggregation of CNDs in a complex physiological environment. We already reported how the surface functionalization can sensibly reduce the CND agglomeration in cell culture media, thus increasing the cellular uptake.^[^ ^12^ ^]^ In addition to their capacity to be efficiently internalized in cells, their high photostability and NIR emission also make MCNDs potential nanoprobes for fluorescence imaging in vitro and in vivo.

### 2.8 Targeted Cancer Cell Uptake and Fluorescence Imaging of MCNDs

**Figure 6:**
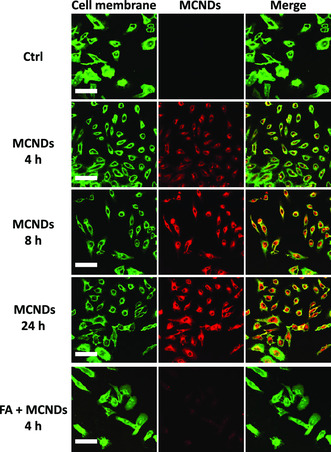
FR‐mediated targeted internalization of MCNDs in cancer cells in vitro. Confocal microscopy images showing the internalization of MCNDs into HeLa cells at different time points (4, 8, 24 h). FA receptor inhibition tests were performed by preincubating the cells with a saturated FA solution (bottom row). In green, membranes stained with Cell Mask (excitation channel: 488 nm, emission: 500−550 nm); in red, MCNDs (excitation channel: 405 nm, emission: 665−715 nm). The scale bar is 100 μm.

Encouraged by the good optical performance and biocompatibility of MCDNs, we next investigated the targeted imaging of cancer cells, that is, HeLa cells. FR is frequently overexpressed on the surface of cancer cells; consequently, FA has been widely used as a targeting ligand for the delivery of therapeutics.^[^ ^34^ ^]^ To evaluate the specific cell targeting of our nanoparticles, MCNDs (at 100 μg mL^−1^) were incubated with HeLa cells (during 4, 8, or 24 h) for confocal fluorescence imaging. MCND‐treated cells showed high intracellular ﬂuorescence signals after 4 h incubation and increased in a time‐dependent manner (**Figure** **6**), thereby confirming the efficient internalization of our nanoparticles into the cytoplasm of HeLa cells. The specificity of FA–FR interaction was further demonstrated by the inhibition of MCND internalization upon addition of an excess of FA as a binding competitor during incubation with HeLa cells (Figure 6). These results demonstrate that MCNDs are able to bind to FR overexpressed on tumor cells and are further efficiently internalized into the cells by a receptor‐mediated endocytosis mechanism.

### 2.9 PDT/PTT of MCNDs in Vitro

**Figure 7:**
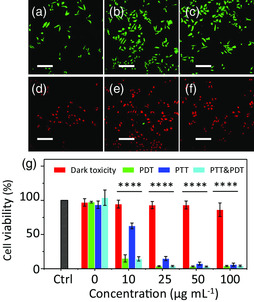
PDT/PTT efficacy of MCNDs in vitro. a–f) Fluorescence images of calcein AM‐/PI‐stained HeLa cells exposed to different conditions: a) Ctrl, b) 660 nm laser only (0.1 W cm^−2^), c) 808 nm laser only (2 W cm^−2^), d) 100 μg mL^−1^ of MCNDs + laser (808 nm, 2 W cm^−2^, PTT), e) 100 μg mL^−1^ of MCNDs + laser (660 nm, 0.1 W cm^−2^, PDT), f) 100 μg mL^−1^ of MCNDs + laser (808 nm, 2 W cm^−2^, PTT) and 660 nm laser (0.1 W cm^−2^, PDT). In green, live cells stained with calcein AM (excitation channel: 488 nm, emission: 500−550 nm); in red, dead cells stained with PI (excitation channel: 488 nm, emission: 620−720 nm). The scale bar is 300 μm. g) Relative viability of HeLa cells incubated with various concentrations of MCNDs in the dark or upon irradiation by a 660 nm laser at 0.1 W cm^−2^ and an 808 nm laser at 2 W cm^−2^ for 10 min. Data are the mean ± sd of three independent experiments (two‐way ANOVA with Bonferroni t‐test: ****p < 0.0001).

We then studied the effects of the bimodal PDT/PTT anticancer therapy of MCNDs in vitro. Calcein AM/propidium iodide (PI) assay was used to differentiate live and dead HeLa cells. In the laser‐only (660 nm at 0.1 W cm^−2^ or 808 nm at 2 W cm^−2^) treated groups, all cells displayed green fluorescence, meaning that cells remained alive under light irradiation (**Figure** **7**a–c). Instead, after incubation with 100 μg mL^−1^ of MCNDs, all cells were destroyed upon 808 nm laser irradiation (2 W cm^−2^, 10 min) for PTT (Figure 7d). Similarly, upon 0.1 W cm^−2^ of 660 nm laser irradiation for PDT, all cells were killed, as indicated by the homogeneous red ﬂuorescence (Figure 7e). In the combined PTT/PDT group, all cells were first irradiated with the 808 nm laser (2 W cm^−2^, 10 min) and then irradiated with the 606 nm laser (0.1 W cm^−2^, 10 min). As shown in Figure 7f, all cells seemed to be dead, as shown with the red PI ﬂuorescence. These results suggest that the MCNDs can generate ^1^O_2_ and produce heat to efficiently kill cells by PDT and PTT, respectively. Subsequently, to quantify these observations, we evaluated the cytotoxic impact and therefore the PDT and PTT efficacy, of NIR MCNDs using the cell counting kit‐8 (CCK‐8) assay. As shown in Figure 7g, the survival of HeLa cells treated with different doses of MCNDs, in the absence of laser irradiation, was not affected. This confirms again the low cytotoxicity and good biocompatibility of NIR MCNDs. In the PTT‐treated group, the percentage of live cells diminished with increasing concentrations of MCNDs. Interestingly, in the PDT‐treated group, the cell viability drastically decreased, already starting at low MCND concentration (Figure 7g). Indeed, ≈90% mortality was induced at 10 μg mL^−1^, reaching ≈99% at 25 μg mL^−1^. The remarkable PDT/PTT efficacy can be attributed to efficient targeting and penetration of MCNDs into HeLa cells. The resulting intercellular ROS and heat production can efficiently destroy cancer cells.

### 2.10 Biodistribution and in vivo Imaging of MCNDs

**Figure 8:**
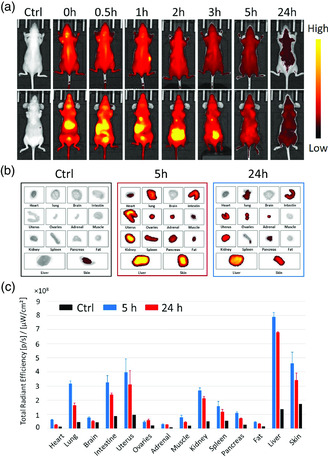
In vivo fluorescence imaging and biodistribution of MCNDs. a) Real‐time in vivo NIR fluorescence images of nude mice at various time points (0, 0.5, 1, 2, 3, 5, 24 h) after intravenous injection of MCNDs (excitation: 640 ± 15 nm, emission: 680 ± 15 nm) (one representative mouse out of 3, top: dorsal side images, bottom: ventral side images). b) Ex vivo NIR fluorescence images of the major organs harvested at 0, 5, and 24 h after intravenous injection of MCNDs (one representative mouse out of 3). c) Average fluorescence intensity of the organs at 0, 5, and 24 h postinjection (*n* = 3 mice, mean ± SEM).

Finally, assessing the biodistribution of nanoparticles into the vital organs is fundamental in view of in vivo biomedical applications. The covalent linkage of Ce6 endowed CNDs with in vivo fluorescence imaging performance. To monitor their organ biodistribution, MCNDs (200 μL, 1 mg mL^−1^ in 0.9% NaCl) were intravenously (i.v.) injected into nude mice, which showed no clinical signs within 24 h after the injection. Blood was collected 5 h postinjection and a strong fluorescence signal could be detected in the plasma (Figure S9, Supporting Information), indicating that MCNDs displayed a long circulation time. The fluorescence images of the mice were acquired at 0, 0.5, 1, 2, 3, 5, and 24 h (**Figure** **8**a). A strong signal was observed in the skin immediately after the i.v. injection (visible on the ventral‐ and dorsal‐side images), indicating a quick diffusion of the nanoparticles. An accumulation was also observed in the liver and bladder just after the injection (ventral‐side images), and then in the gallbladder and intestines, which is indicative of a hepatobiliary and renal urinary dual elimination route. These signals decreased gradually over time but without disappearing completely between 5 and 24 h. On the dorsal side images, no signal could be observed in the kidneys, most probably because it was likely masked by the strong signal from the skin. After 24 h, the organs were collected for fluorescence imaging (Figure 8b,c). The ex vivo fluorescence signals confirmed the dual hepatobiliary and renal elimination pathway of MCNDs, as strong signals could be detected in the liver, intestines, and kidney at 5 and 24 h postinjection. Fluorescent signals were also observed in the skin, uterus, lung, spleen, pancreas, muscle, heart, fat, ovaries, and adrenals (likely due to a facilitated MCND extravasation because of their nanoscale size). These signals decreased with time, but significant residual signals were still observed 24 h after injection.

## 3 Conclusion

In summary, a flexible method of double covalent functionalization of CNDs has been successfully developed based on a double amidation approach. A high loading of the poorly soluble Ce6 to the hydrophilic CNDs remarkably improved the drug solubility in physiological conditions while maintaining NIR photosensitizing properties. The resulting MCNDs own the advantages of a well‐defined structure, low size, precise drug loading, high water dispersibility, NIR fluorescence emission, and good photodynamic/photothermal efficiency upon NIR laser irradiation. In vitro tests indicated that MCNDs own an efficient FR‐mediated targeting capacity to penetrate into cancer cells, leading to cell killing at a low dose under NIR laser irradiation. They displayed limited cytotoxic and inflammatory effects and a long blood circulation period. It is worth noting that they could be excreted through a dual hepatobiliary and renal elimination route, while some were still present in the liver, intestines, and kidney 24 h postinjection.

The covalent modification of CNDs for the delivery of hydrophobic PSs have the following advantages: 1) the amount of functional ligands could be accurately controlled by adjusting the ratio of functional ligands and the reaction conditions; 2) CNDs can prevent the aggregation of PSs and improve their stability and solubility in biological media; 3) compared with noncovalent conjugates, the covalent PS−CND can remain stable in the bloodstream, which could not only prolong the blood circulation half life of the PS, but could also avoid release before reaching the target area; and 4) CNDs can be efficiently eliminated after completing their mission, thus minimizing potential long‐term toxicity.

Our study reinforces the potential of CNDs for the safe and efficient delivery of hydrophobic PSs. Owing to their high dispersibility and the versatility of the double functionalization approach, CNDs could be designed to prepare multifunctional nanomedicines for the treatment of various diseases, including cancer and autoimmune diseases. For instance, other targeting ligands and drugs, such as peptides, aptamers, sugars, or anticancer drugs, could be conjugated onto CNDs. We hope our strategy for covalent multifunctionalization of CNDs will be inspiring for the design of second generation of “all‐in‐one” cancer theranostic reagents with natural capabilities in location, imaging, and treatment.

## 4 Experimental Section

4.1

4.1.1

##### Chemicals and Materials

All chemicals and materials used were commercially available and purchased from different commercial suppliers and used as received. All chemicals were of analytical grade or of the highest purity available. Ultrapure water with resistivity of 18.2 MΩ was obtained from a Milli‐Q integral Pure/Ultrapure Water Production unit. In particular, FA, 1‐ethyl‐3‐(3‐dimethylaminopropyl)carbodiimide hydrochloride (EDC·HCl) and *N*‐hydroxysuccinimide (NHS) were purchased from Alfa Aesar. Ce6 and dihydrorhodamine‐123 (DHR 123) were purchased from Sigma‐Aldrich. 9,10‐anthracenediyl‐bis(methylene) dimalonic acid (ABDA) and MB were purchased from Across Organics. Calcein AM/PI kit was purchased from Sigma‐Aldrich. Compound 1 was synthesized following a protocol reported in a previous work.^[^ ^12^ ^]^ LC/MS analyses were performed on ThermoFisher Finnigan 6 LCQ Advantage Max. ^1^H NMR spectra were recorded on Bruker DPX 300 instrument. The peak values were obtained as ppm (*δ*) and referenced to the solvent. The resonance multiplicity was indicated as s (singlet), d (doublet), t (triplet), dd (doublet of doublet), and m (multiplet). Centrifugation and bath sonication were performed using an Eppendorf centrifuge 5804R and an Elmasonic P sonicator, respectively. The dialysis was performed using membranes with MWCO of 1 and 2 kDa from Spectrum Laboratories Inc.

##### Instruments for the Characterization of Materials

HPLC was performed using a Nucleosil 100‐5 Waters C18 reverse‐phase HPLC column and a Waters Alliance e2695 separation module. The column was used with a 1.2 mL min^−1^ flow rate of a gradient from 0 to 100% of B (A = H_2_O/0.1% trifluoroacetic acid [TFA]; B = CH_3_CN/0.08% TFA) for 20 min. Fluorescence steady‐state spectra were recorded via a Fluorolog FL3‐22 (Horiba Jobin Yvon) spectrometer using a swig xenon 450 W lamp. UV−vis spectra were recorded using a VARIAN 5000 spectrometer. The morphology of the samples was studied by TEM (JEM‐2010F, JEOL, Japan). Thermogravimetric analyses were performed on a TGA1 (Mettler Toledo) with a ramp of 10 °C min^−1^ under N_2_ using a flow rate of 50 mL min^−1^ and platinum pans. Photodynamic tests were executed with a 660 nm laser (Changchun new industries optoelectronics tech.co., ltd). For the photothermal studies, we used an 808 nm laser diode system from Roithner Lasertechnik (LOSBLD‐0808‐2W‐C/P) and a thermal imaging camera from Flir. The DLS measurements were done with a Zetasizer Nano ZS (Malvern, UK). XPS was performed using Thermo Scientific KAlpha X‐ray spectrometer with a mono X‐ray source Al Kα excitation (1486 eV). Binding energy calibration was based on C 1*s* at 284.7 eV. Photoluminescence quantum yield was performed on an FLS 1000, Edinburgh Instruments Ltd., and measured by a Hamamatsu Quantaurus‐QY integrating sphere under an air‐equilibrated condition using an empty quartz tube as a reference.

##### Synthesis of CNDs

CNDs were obtained from a method from our previous reports.^[^ ^13^ ^]^ In brief, CNDs were prepared by hydrothermal treatment of PPA. 20 mg of PPA were dispersed in 40 mL of NaOH solution (1 mM). The mixture was treated ultrasonically for 3 h and then transferred into an autoclave and heated at 220 °C for 24 h. After cooling to room temperature, the CNDs were collected through filtering with 0.22 μm membranes (Millipore, PTFE) three times. The filtrate was dialyzed (1 kDa membrane) against distilled water for 2 days to remove the residual NaOH. After lyophilization, a red powder was obtained.

##### Preparation of FA−TEG−NH_2_ (**4**)

This compound was prepared according to our previous work.^[^ ^12^ ^]^ Briefly, FA (440 mg, 1 mmol) and compound **1** (298 mg, 1.2 mmol) in DMSO reacted for 20 h in the presence of EDC·HCl /NHS/TEA (2 mmol/2 mmol/4 mmol). After purification by column chromatography (chloroform/EtOH/acetone/NH_3_·H_2_O (30%) = 2:2:2:1), compound **2** (180 mg, 36%) was obtained. Compound **2** (50 mg) was deprotected in 1 mL TFA, giving compound **4** as a yellow solid (35 mg, 82%). ^1^H NMR (400 MHz, DMSO‐d_6_) *δ* 8.65 (s, 1H), 8.22–8.01 (m, 1H), 7.97–7.78 (m, 1H), 7.65 (d, *J* = 7.8 Hz, 2H), 7.17–6.82 (m, 3H), 6.65 (d, *J* = 7.8 Hz, 2H), 4.50 (s, 2H), 4.35–3.97 (m, 1H), 3.80–3.45 (m, 6H), 3.20 (d, *J* = 4.5 Hz, 4H), 3.10–2.91 (m, 3H), 2.87–2.70 (m, 1H), 2.59 (s, 1H), 2.35–2.11 (m, 2H), 2.11–1.85 ppm (m, 2H). LC‐MS (ESI): *m/z* calculated for C_25_H_23_N_9_O_7_: 571.25, found 572.18 [M + H]^+^. HPLC (*t*_R_ = 6 min over 20 min of 0.6 mL min^−1^ mobile phase (90% acetonitrile and 10% water)).

##### Preparation of Ce6−TEG−NHBoc (**3**)

Ce6 (100 mg, 0.17 mmol), EDC·HCl (63 mg, 0.34 mmol), NHS (40 mg, 0.34 mmol), and triethylamine (TEA, 100 μL, 0.68 mmol) were dissolved in 10 mL anhydrous dichloromethane and then stirred below 0 °C for 4 h. After adding compound **1** (50 mg, 0.2 mmol), the reaction solution was stirred at room temperature for 24 h. Afterward, the reaction mixture was condensed by rotary evaporation and then purified by thin layer chromatography (TLC) using a mixture of dichloromethane/methanol (5:1, v/v). Then, the individual band was scraped. Using flash chromatography to remove silica, compound **3** was obtained as a dark solid (34 mg, 24%). The ^1^H NMR spectrum of Ce6−TEG−NHBoc is shown in Section S2.

^1^H NMR (400 MHz, MeOD) *δ* 9.41 (s, 1H), 8.99 (s, 1H), 8.95 (s, 1H), 7.54 (dd, *J* = 17.7, 11.6 Hz, 1H), 5.89 (d, *J* = 17.9 Hz, 1H), 5.82–5.62 (m, 2H), 5.37 (d, *J* = 18.2 Hz, 1H), 4.75–4.46 (m, 2H), 3.52 (s, 3H), 3.46–3.34 (m, 4H), 3.28–3.12 (m, 7H), 2.84 (s, 1H), 2.82–2.74 (m, 4H), 2.69 (s, 4H), 2.56 (s, 1H), 2.49–2.31 (m, 5H), 1.74 (d, *J* = 6.9 Hz, 3H), 1.45 (t, *J* = 7.1 Hz, 3H), 1.39–1.32 (m, 2H), 1.28 (s, 9H). LC‐MS (ESI): *m/z* calculated for: C_45_H_58_N_6_O, 826.43, found 827.42 [M + H]^+^. HPLC (*t*_R_ = 15.6 min over 20 min of 0.6 mL min^−1^ mobile phase [90% acetonitrile and 10% water]).

##### Preparation of Ce6−TEG−NH_2_ (**5**)

In a round‐bottomed flask, 34 mg of compound **3** was mixed with 1 mL of TFA and stirred for 2 h at room temperature. TFA was removed under reduced pressure. The obtained dark solid was washed with cyclohexane three times and dried in vacuum. Compound **5** (30 mg, >95%) was obtained as a dark solid and stored in the dark below 20 °C for further use. The ^1^H NMR spectrum of compound **5** is shown in Section S2.

^1^H NMR (400 MHz, MeOD) *δ* 10.17 (s, 1H), 9.96 (s, 1H), 9.43 (s, 1H), 8.18 (dd, *J* = 17.5, 11.9 Hz, 1H), 6.34 (dd, *J* = 33.4, 14.7 Hz, 2H), 5.82–5.34 (m, 2H), 4.79–4.69 (m, 1H), 4.61 (d, *J* = 10.0 Hz, 1H), 3.98–3.83 (m, 2H), 3.68 (d, *J* = 15.4 Hz, 10H), 3.61–3.41 (m, 7H), 3.28 (s, 2H), 3.24–3.12 (m, 3H), 3.10 (s, 2H), 2.83–2.68 (m, 1H), 2.62 (s, 2H), 2.52–2.39 (m, 1H), 2.37–2.22 (m, 1H), 1.84 (d, *J* = 6.6 Hz, 3H), 1.71 (s, 1H), 1.60 (t, *J* = 7.1 Hz, 3H). LC‐MS (ESI): *m/*z calculated for: C_40_H_50_N_6_O_7_, 726.37, found 727.37 [M + H]^+^. HPLC (*t*_R_ = 12.7 min over 20 min of 0.6 mL min^−1^ mobile phase [90% acetonitrile and 10% water]).

##### Conjugation of FA−TEG−NH_2_ to CNDs

A suspension of CNDs (10 mg) in DI water (10 mL) was sonicated in an ice bath for 10 min. NHS (20 mg), EDC·HCl (64 mg), and TEA (48 μL) were added to the mixture at room temperature. The suspension was stirred for 2 h to activate the COOH groups on the surface of CNDs. Afterward, a solution of **4** (10 mg) in DMSO (0.5 mL) was carefully added and the mixture was stirred for another 20 h at room temperature. Unbound compound **4** was removed by dialysis (1 kDa membrane) against DI water for 2 days. After lyophilization, a red solid (FCNDs) was obtained.

##### Conjugation of Ce6−TEG−NH_2_ to FCNDs

A suspension of FCNDs (10 mg) in DI water (10 mL) was sonicated in an ice bath for 10 min. NHS (20 mg), EDC·HCl (64 mg), and TEA (48 μL) were added to the mixture at room temperature. The suspension was stirred for 2 h. Afterward, a solution of **5** (10 mg) in DMSO (0.5 mL) was carefully added, and the mixture was stirred for another 20 h at room temperature. Unbound compound **5** was removed by dialysis (2 kDa membrane) against DI water for 2 days in the dark. After lyophilization, a dark red solid (MCNDs) was obtained.

##### Calculation of ROS

DHR 123 was used to measure the ROS generation in solution. The oxidation of DHR 123 by ROS resulted in the formation of fluorescent rhodamine 123. In a typical assay, MCNDs (25 μg mL^−1^) were added to an aqueous solution of DHR 123 (20 nM). Then, the mixture was irradiated using a 660 nm laser (0.1 W cm^−2^) for 0−20 min, and the emission intensity at 530 nm was measured upon excitation at 485 nm.

##### Calculation of Singlet‐Oxygen Quantum Yield^[^ ^26^, ^29^ ^]^

To calculate the singlet‐oxygen quantum yield, ABDA was used as the ^1^O_2_ indicator, and MB was used as the standard reference. In brief, 20 μL of ABDA solution (1 mg mL^−1^) was added into 2 mL of CNDs (25 μg mL^−1^) and MB (1.5 μM) solution. The mixtures were exposed to 660 nm laser with a power density of 0.1 W cm^−2^. The absorption of ABDA at 400 nm was recorded at various irradiation times to obtain the decay rate of the photosensitizing process. The singlet‐oxygen quantum yield of MCNDs (*η*_MCNDs_) in water was calculated according to

(1) $$\eta_{\text{MCNDs}}=\eta_{\text{MB}}\times \frac{K_{\text{MCNDs}}}{K_{\text{MB}}}\times \frac{I_{\text{MB}}}{I_{\text{MCNDs}}}$$

where *K*_MCNDs_ and *K*_MB_ are the decomposition rate constants of ABDA by the MCNDs and MB, respectively. *I*_MB_ and *I*_MCNDs_ represent the light absorbed by the MCNDs and MB at 660 nm, respectively, and $\eta_{\text{MB}}$ refers to the ^1^O_2_ quantum yield of MB in water ($\eta_{\text{MB}}$ = 0.52). From Figure 3c, $K_{\text{MB}}$ is 0.22 and $K_{\text{MCNDs}}$ is 0.02. From Figure S8, Supporting Information, *I*_MB_ = 0.11 and *I*_MCNDs_ = 0.03. Therefore, the ^1^O_2_ quantum yield of MCNDs $\eta_{\text{MCNDs}}$ corresponds to 0.173.

##### Calculation of the Photothermal Conversion Efficiency

To measure the photothermal conversion efficiency, 0.5 mL of an MCND aqueous solution in a quartz cuvette was irradiated by an 808 nm NIR laser for 10 min. The real‐time thermal imaging of the quartz was recorded every 30 s by a Fluke thermal camera (Ti‐400).

Photothermal conversion efficiency (*η*) was calculated by Roper's method.^[^ ^32^, ^33^ ^]^ The temperature change of the MCND dispersion (400 μg mL^−1^) was recorded under the continuous 808 nm laser irradiation until reaching a steady‐state temperature. *η* was calculated via Equation (2).

(2) $$\eta =\frac{hs\left(T_{\text{max}}-T_{\text{sur}}\right)-Q_{\text{Dis}}}{I\left(1-10^{-A_{808}}\right)}$$

where *h* is the heat transfer coefficient, *s* is the surface area of the container, $T_{\text{max}}$ is the equilibrium temperature (46.4 °C), $T_{\text{sur}}$ is the ambient temperature of the environment (23.1 °C), *I* is incident laser power (1000 mW), *Q*_Dis_ is the heat dissipation from the light absorbed by the quartz sample cell [*Q*_Dis_ = (5.4 × 10^−4^) *I* = 0.54 mW], and *A*_808_ is the absorbance of MCNDs at 808 nm (0.2). The value of *hs* was derived according to Equation (3).

(3) $$\tau =\frac{m_{D}c_{D}}{hs}$$

where *τ* is the sample system time constant and *m*_D_ and *c*_D_ are the mass (0.5 g) and heat capacity of water (4.2 J/(g·°C)) of the deionized water used as solvent, respectively. To obtain *τ*, a dimensionless driving force temperature *θ* was introduced.

(4) $$\theta =\frac{T-T_{\text{sur}}}{T_{\text{max}}-T_{\text{sur}}}$$

After getting the curve of *t *= −*τ* ln*θ* (Figure 3f, *τ* was determined to be 507 s). According to Equation (3), *hs* was calculated to be 4.14 mW·°C^−1^. Then, according to Equation (2), *η* was calculated to be 0.259.

##### Cell Culture

The RAW 264.7 cell line and HeLa cells were cultured in Dulbecco's Modified Eagle Medium (DMEM, Corning) supplemented with 10% fetal bovine serum (FBS, Dutscher), 10 μg mL^−1^ gentamycin, 10 mM HEPES, and 0.05 mM β‐mercaptoethanol (Pan Biotech), in an atmosphere of 5% CO_2_ and at 37 °C. The cells were passaged after reaching 90% confluence and then detached with a cell scrapper and subcultured in T‐75 flasks. The cells were regularly tested for mycoplasma contamination.

##### Macrophage Viability and Cytokine Production

RAW 264.7 cells (0.5 × 10^6^) were seeded in 12‐well plates (Falcon) in 400 μL complete medium for 24 h. Then, the medium was removed and 400 μL of CNDs or MCNDs were added at final concentrations of 2, 5, 20, and 50 μg mL^−1^ in complete medium. LPS (*Escherichia coli* 0111:B4, Sigma, 1 μg mL^−1^) were used as a positive control for cytokine secretion. After 24 h, the supernatants were collected for cytokine measurements, and cells were gently detached using a scraper. The cell viability was assessed by flow cytometry using FVD 780 (Fixable Viability Dye eFluor780, ThermoFisher) and a Gallios flow cytometer (Beckman Coulter). IL‐6, IL1‐β, and TNF‐α were detected by ELISA as previously described.^[^ ^35^ ^]^

##### Macrophage Imaging

RAW 264.7 cells were harvested from T‐75 mL cell culture flasks. These cells were then centrifuged for 5 min at 3000 rpm. The supernatant was discarded, and 5 mL DMEM complete media were added to the cell pellet before cell counting was performed. ≈1 × 10^4^ cells per well were seeded in an 8‐well chamber slide (Thermo Scientific) and cultured until it reached 80% confluence. The medium was then replaced with a medium containing 100 μg mL^−1^ of CNDs or MCNDs. After incubation for 0, 4, and 8 h, 2 μL of CellMask was added to each well and incubated for 10 min at 37 °C. Confocal live images were then obtained with a Zeiss Axio Observer Z1 spinning disk confocal microscope equipped with a 63× oil objective. z‐Stacking was recorded with 0.3 μm interplanar distance. The fluorescence signal from CellMask was obtained using a 488 nm laser excitation and recorded in the green channel (500−550 nm), whereas CNDs and MCNDs were recorded using a 405 nm laser excitation in the NIR channel (665−715 nm). The images were then treated with ImageJ software.

##### HeLa Targeting and Imaging

HeLa cells (1 × 10^4^ cells/well) were seeded in glass‐bottomed culture dishes and incubated with MCNDs (100 μg mL^−1^) for 0, 8, and 24 h at 37 °C. Cell Mask green from Sigma‐Aldrich was then added to each well and the cells incubated for additional 10 min at 37 °C. To confirm the receptor‐mediated uptake, competition experiments were performed: HeLa cell cultures were pretreated with 50 μL of a saturated FA solution for 1.5 h, prior to adding MCNDs. All confocal images were acquired using a Leica laser scanning confocal fluorescence microscope. The saturated FA solution was prepared according to the following procedure: 10 mg of FA was added to 1 mL PBS and the suspension was sonicated for 10 min. After centrifugation at 10 000 rpm for 20 min, the supernatant was collected.

##### MCND‐Induced PDT/PTT on HeLa Cells in vitro

HeLa cells were incubated with or without MCNDs (100 μg mL^−1^) for 4 h and then irradiated by a 660 nm laser for 10 min. Then cells were costained with calcein AM and PI (Sigma‐Aldrich) for 30 min, washed with PBS, and imaged by a Leica laser scanning confocal microscope. The CCK‐8 (Sigma‐Aldrich) assay was also carried out to quantify cell viability to further confirm the phototherapy efficacy of MCNDs. HeLa cells (1 × 10^4^ cells/well) were seeded in 96‐well plates and incubated with different concentrations (0, 10, 25, 50, 100 μg mL^−1^) of MCNDs at 37 °C for 24 h under the same conditions and then irradiated by 660 nm laser (0.1 W cm^−2^) or 808 nm laser (2 W cm^−2^) for 10 min. All cell media were then replaced with fresh cell media. An amount of 10 μL of CCK‐8 was added to each well, and then the cells were incubated for 1 h at 37 °C with 5% CO_2_. The absorbance at 450 nm was measured by a multimode microplate reader (Synergy H1, BioTek) to calculate the cell survival rate.

##### In vivo Fluorescence Imaging

Animal experimentations were performed in accordance with the institutional guidelines of the European Community (EU Directive 2010/63/EU) for the use of experimental animals and received the approval of the local ethics committee (Cometh38 Grenoble, France) and the French Ministry of Higher Education and Research (reference APAFIS#8854‐20 170 301 314 338 357; Animal Facility approval number: D 3 851 610 001).

For in vivo fluorescence imaging, seven female NMRI nude mice (8‐week‐old, Janvier Labs, Le Genest‐Saint Isle, France) were injected intravenously via the tail vein with 200 μL of MCNDs (1 mg mL^−1^). Mice were anesthetized using air/isoflurane 4% for induction and 1.5% thereafter. In vivo fluorescence imaging was performed using an IVIS Kinetic (PerkinElmer, USA) with 640 ± 15 nm excitation and 680 ± 15 nm emission filters. Whole‐body fluorescence imaging was performed before and at several time points after the intravenous administration of MCNDs. Mice were sacrificed at 5 and 24 h postinjection, and some organs were collected for ex vivo fluorescence imaging. Organs from control mice were also imaged to determine the autofluorescence of each organ.

##### Statistical Analysis

All statistical analyses were performed using GraphPad Prism (GraphPad software). All datasets were analyzed for significance using two‐way ANOVA with Bonferroni posthoc test. All data were addressed as means ± SEM. All data were performed at least in three independent replicates of experiments. No methods were applied to pre‐estimate sample size.

## Conflict of Interest

The authors declare no conflict of interest.

## Data Availability Statement

Data are available upon request to the authors.

## Supporting information

Supplementary Material
