# Supplementary material for: Multifunctional Carbon Nanodots: Enhanced Near‐Infrared Photosensitizing, Photothermal Activity, and Body Clearance
Source: Small Sci. 2021 Dec 5;2(2):2100082. doi: 10.1002/smsc.202100082 (PMC11936047; doi:10.1002/smsc.202100082)

Supporting Information

Multifunctional Carbon Nanodots: Enhanced NIR Photosensitizing, Photothermal activity and Body Clearance

Ding-Kun Ji,[#] Hayet Dali, Shi Guo, Sowmya Malaganahally, Hélène Dumortier, Cécilia Ménard-Moyon, Alberto Bianco[*]

CNRS, Immunology, Immunopathology and Therapeutic Chemistry, UPR 3572, University of Strasbourg, ISIS, 67000 Strasbourg, France

Current address: [#] Institute of Molecular Medicine (IMM), Renji Hospital, School of Medicine, Shanghai Jiao Tong University, Shanghai 200240, China

Correspondance: a.bianco@ibmc-cnrs.unistra.fr

Content list:

S1. Additional Schemes and Figures SI-S10

S2. NMR spectra of Ce6 and Ce6 ligands

SI Additional Schemes and Figure S1-S10

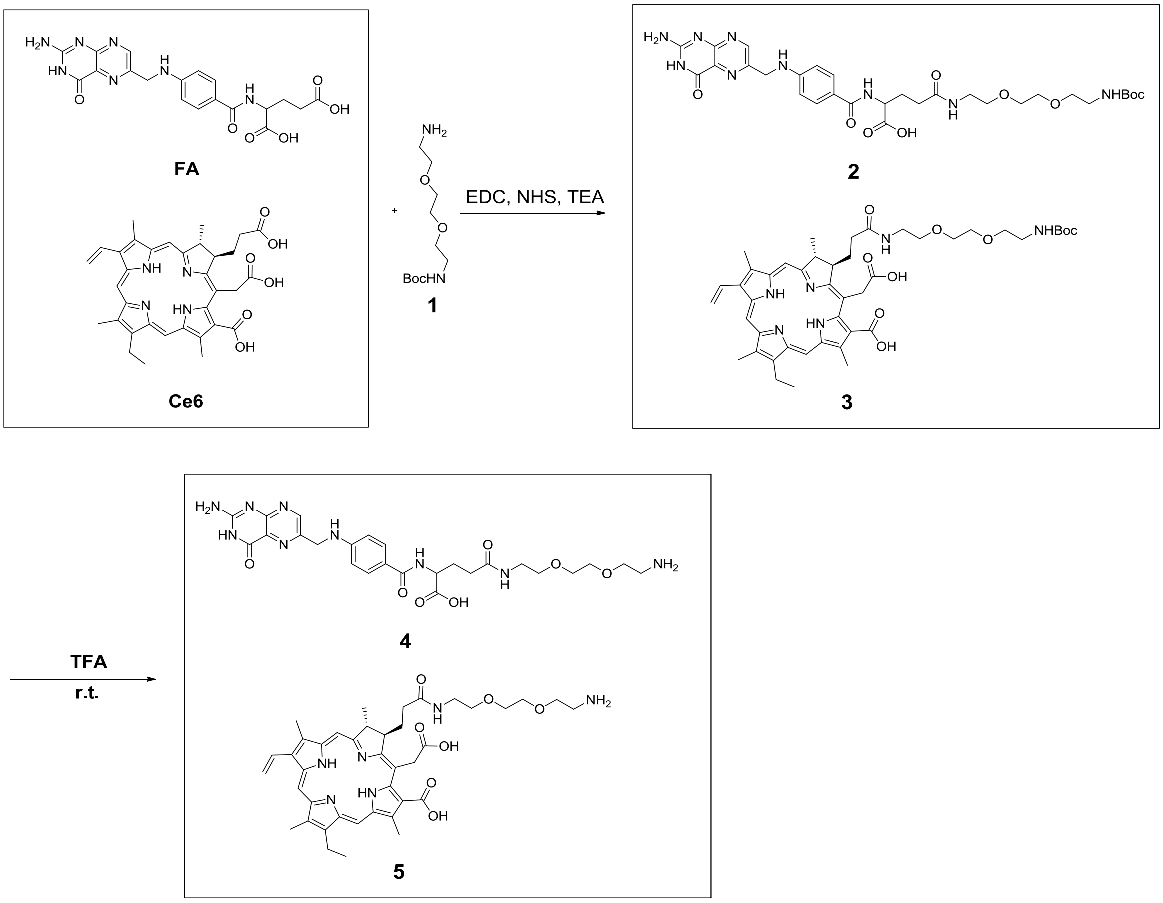

Scheme S1: Synthetic pathway to prepare FA ligand and Ce6 ligand.

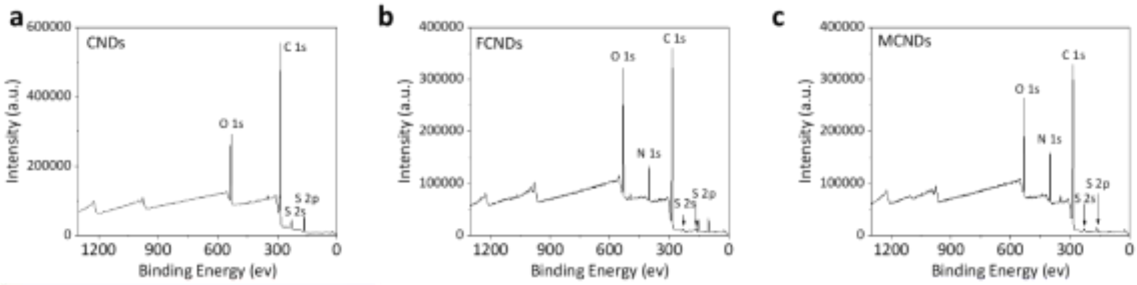

Name	Position	FWHM	Area	At%
C 1s	284.08	2.41	1594594.60	81.66
O 1s	532.08	3.27	801410.67	14.01
S 2p	164.08	2.69	141916.76	4.33

Name	Position	FWHM	Area	At%
C 1s	285.08	2.66	1113104.09	72.66
N 1s	400.08	2.38	194807.64	7.06
O 1s	532.08	2.89	879282.91	19.59
S 2p	164.08	2.83	17587.29	0.68

Name	Position	FWHM	Area	At%
C 1s	285.08	3.05	1159162.62	72.90
N 1s	400.08	2.55	302450.68	10.75
O 1s	532.08	2.95	714638.99	15.34
S 2p	164.08	2.88	31740.24	1.19

Figure S1. The XPS survey spectra and element content of (a) CNDs, (b) FCNDs, (c) MCNDs.

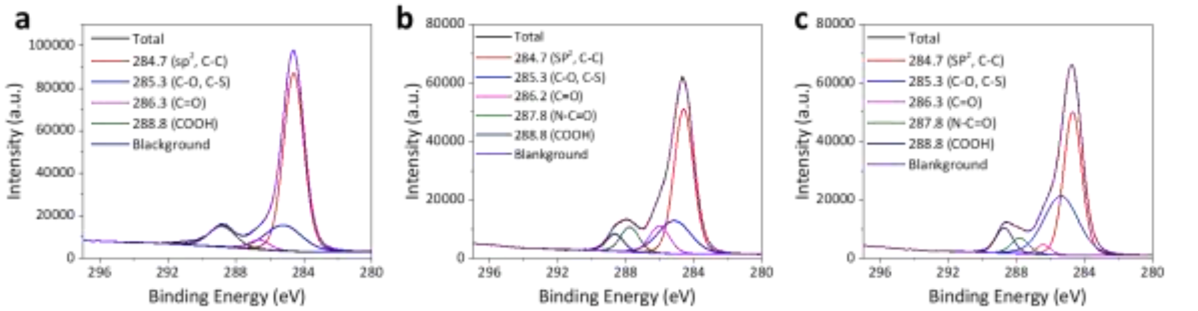

Name	Position	FWHW	Area	At%
C-C	284.7	1.4	130602.21	68.31
C-O, C-S	285.3	2.51	33771.31	17.67
C=O	286.3	1.43	6973.78	3.65
O-C=O	288.8	1.84	19834.00	10.37

Name	Position	FWHM	Area	At%
C-C	284.7	1.33	73524.25	53.86
C-O, C-S	285.3	2.28	28621.51	20.97
C=O	286.3	1.40	14465.04	10.60
N-C=O	287.8	1.28	12099.12	8.86
O-C=O	288.8	1.17	7800.83	5.71

Name	Position	FWHM	Area	At%
C-C	284.7	1.24	67855.21	47.65
C-O, C-S	285.3	2.36	52633.30	36.96
C=O	286.3	0.95	3532.97	2.48
N-C=O	287.8	1.23	7728.95	5.08
O-C=O	288.8	1.16	11144.59	1.83

Figure S2. The high resolution C 1s and chemical bond content of (a) CNDs, (b) FCNDs, (c) MCNDs.

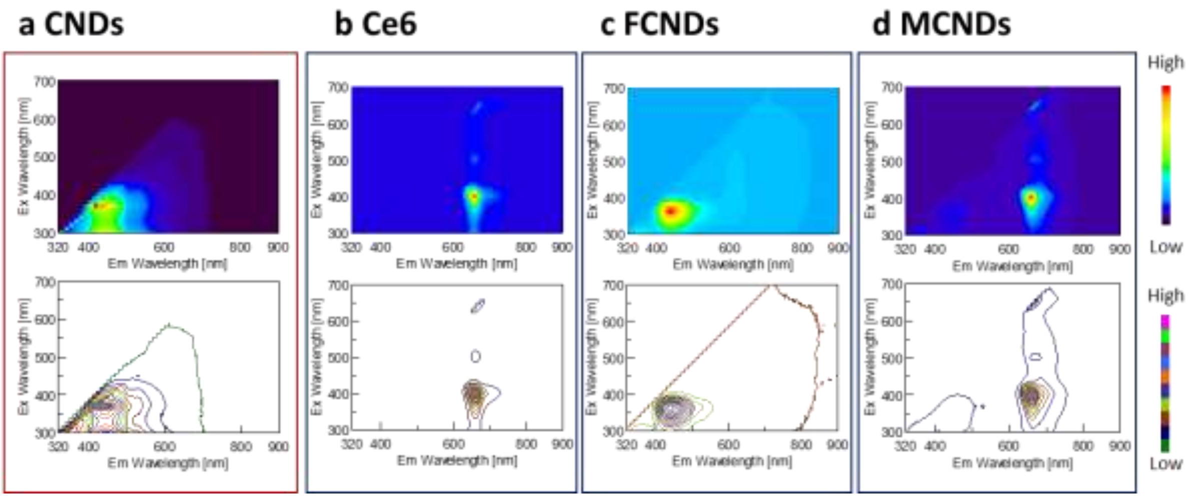

Figure S3. Fluorescence spectroscopy of CNDs, Ce6, FCNDs, and MCNDs.

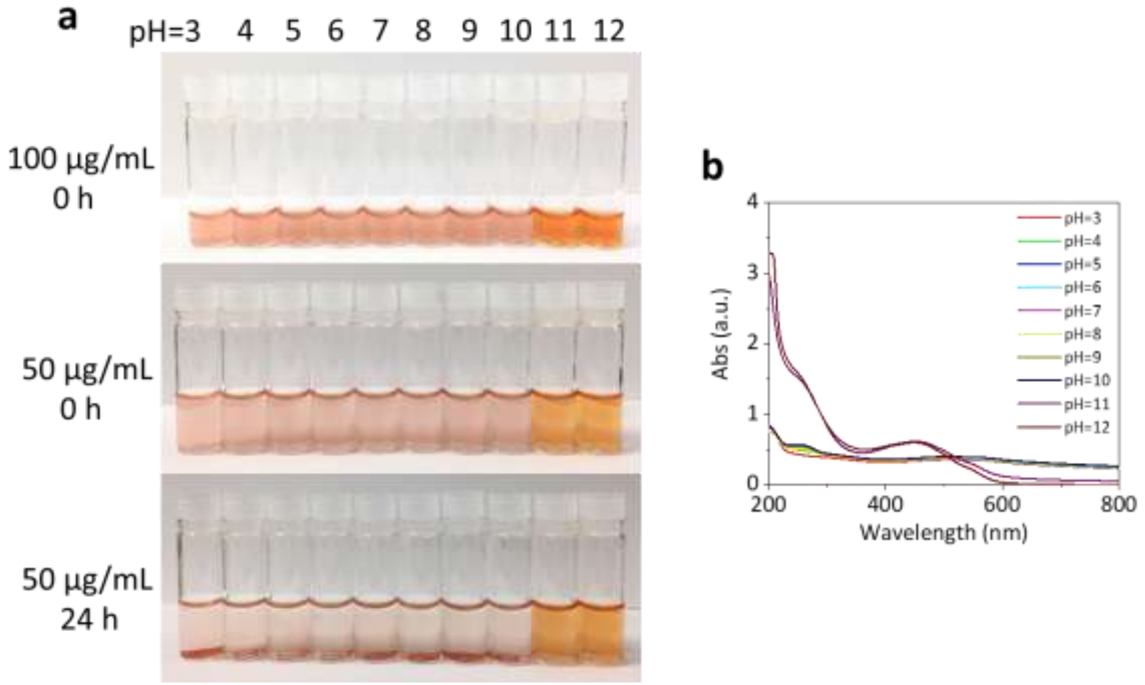

Figure S4. (a) Aqueous dispersions of CNDs at different pH and (b) corresponding UV-Vis spectra.

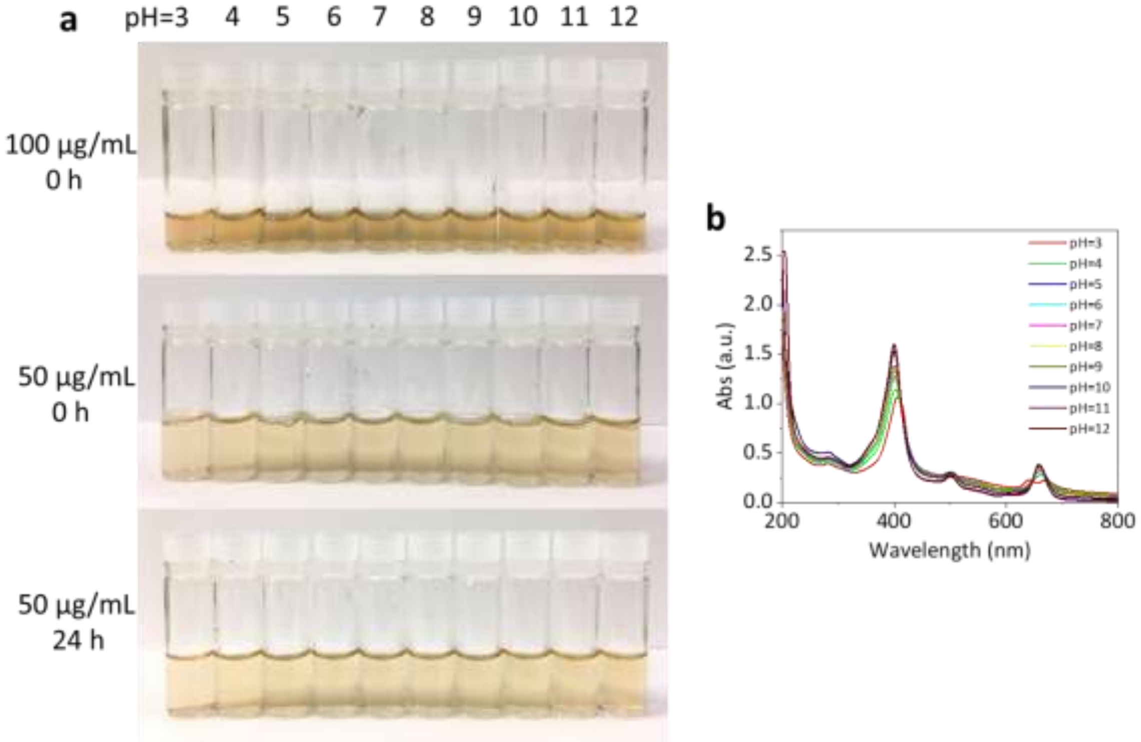

Figure S5. (a) Aqueous dispersions of MCNDs at different pH and (b) corresponding UV-Vis spectra.

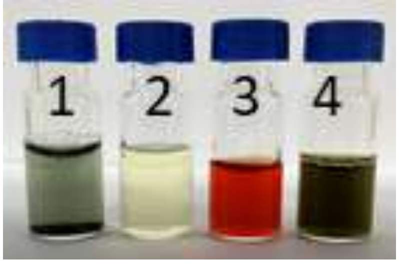

Figure S6. Digital pictures of (1) Ce6, (2) FA, (3) CNDs and (4) MCNDs at 1 mg·mL^{-1} in PBS, pH=7.4.

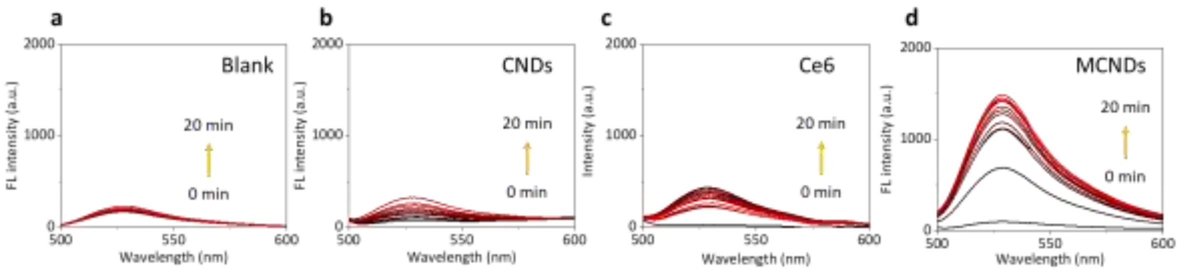

Figure S7. Time-dependent fluorescence titration of DHR 123 (20 nM) without (a) or with (b) CNDs (25 μg·mL^{-1}), (c) Ce6 (5 μg·mL^{-1}), MCNDs (25 μg·mL^{-1}). All measurements were carried out with an excitation of 485 nm under laser irradiation at 660 nm for a period of 20 min.

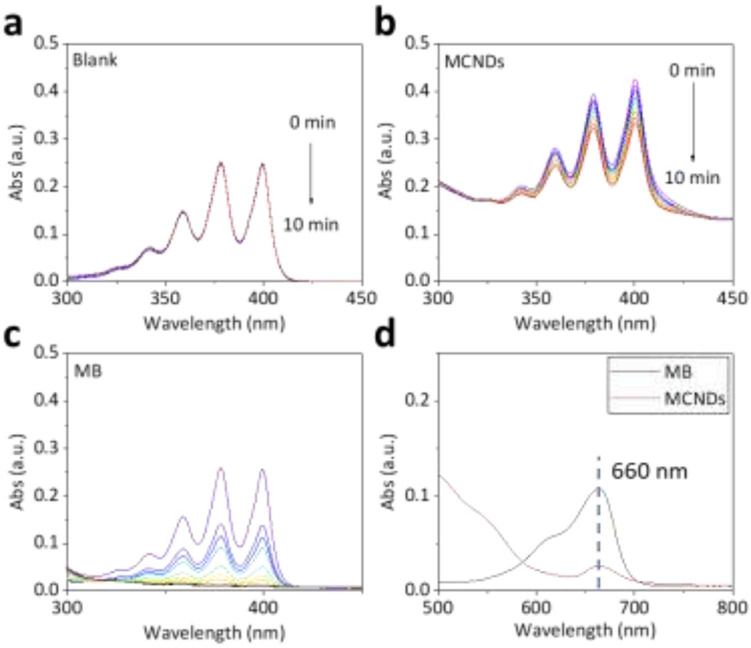

Figure S8. Photodegradation of ABDA with a) DI water, b) MCNDs (25 μg·mL^{-1}) and c) MB (1.5 μM), under 660 nm laser irradiation (0.1 W·cm^{-2}). d) Absorbance of MCNDs and MB at 660 nm.

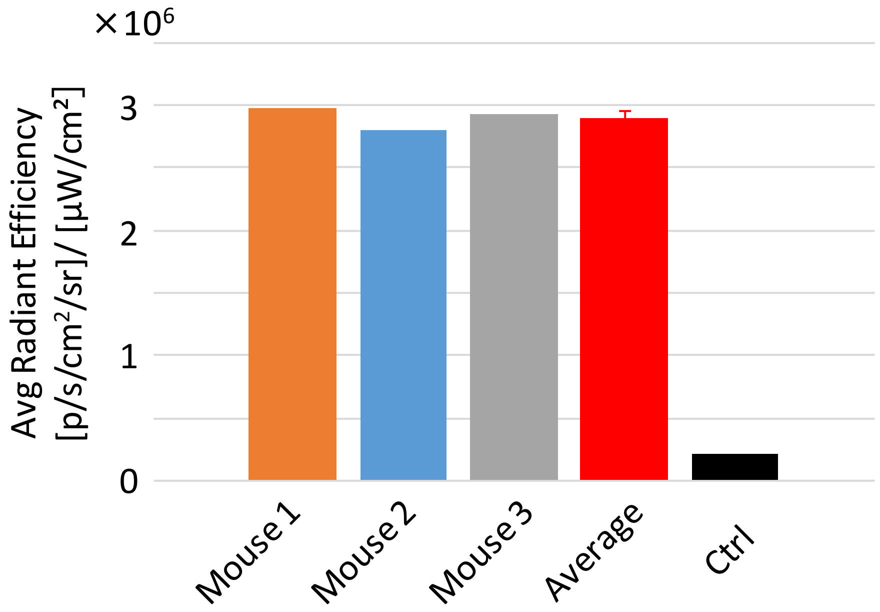

Figure S9. *Ex vivo* fluorescence imaging on 10 µL of plasma after 5 h post-injection. Average fluorescence intensity of the organs at 5 h post-injection (n=3 mice, mean+/- SEM).

S2. NMR spectra of Ce6 and Ce6 ligands

Ce6

1H NMR

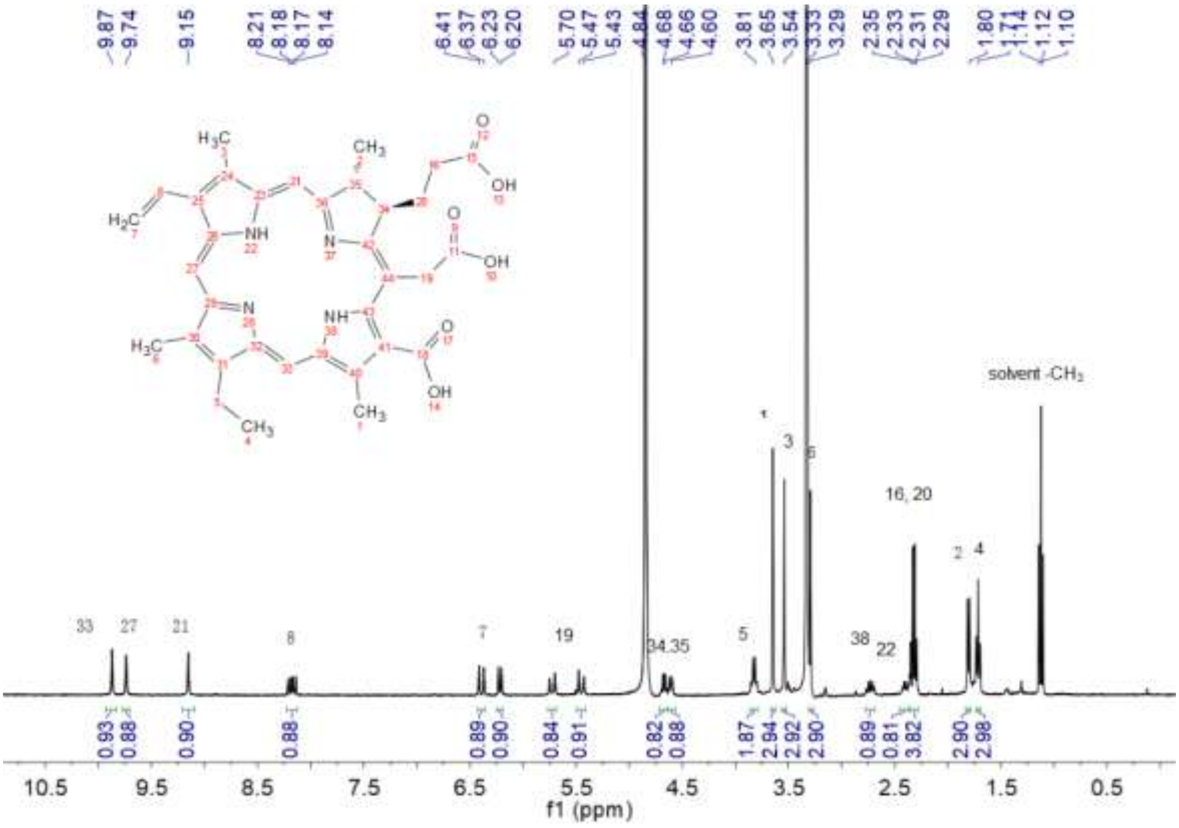

Ce6-TEG-NHBoc

1H NMR

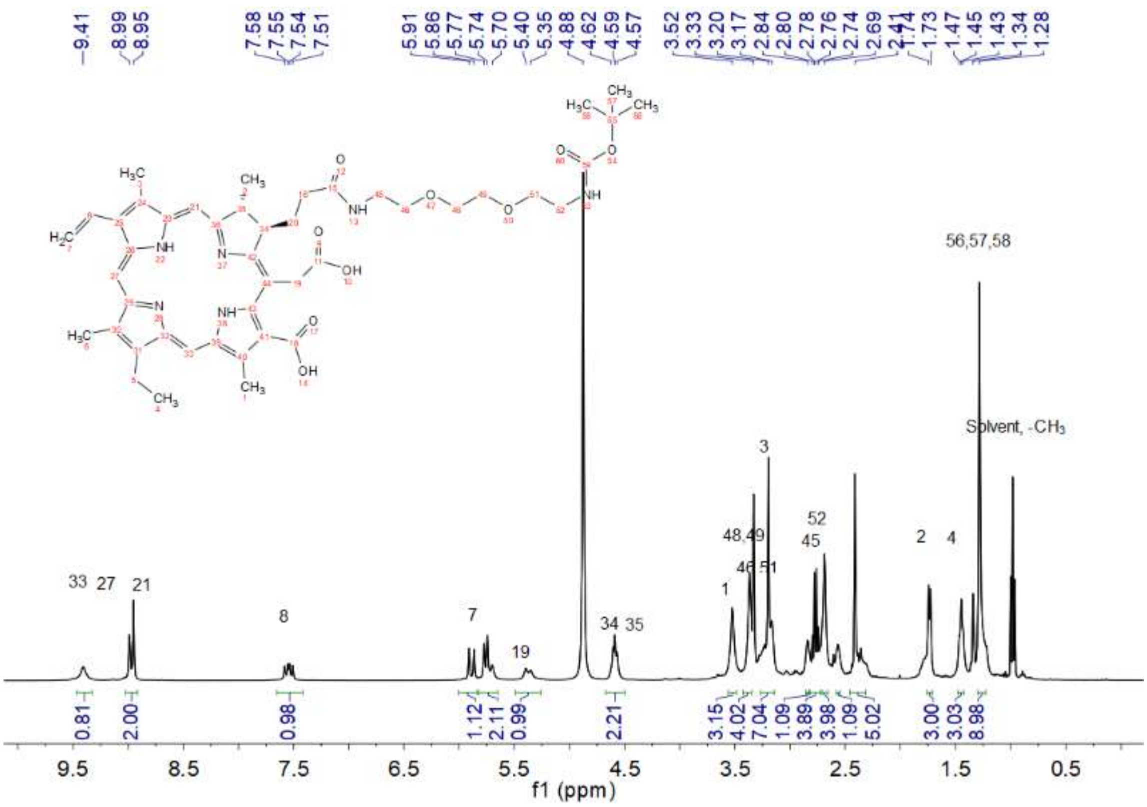

Ce6-TEG-NH$_2$

1H NMR

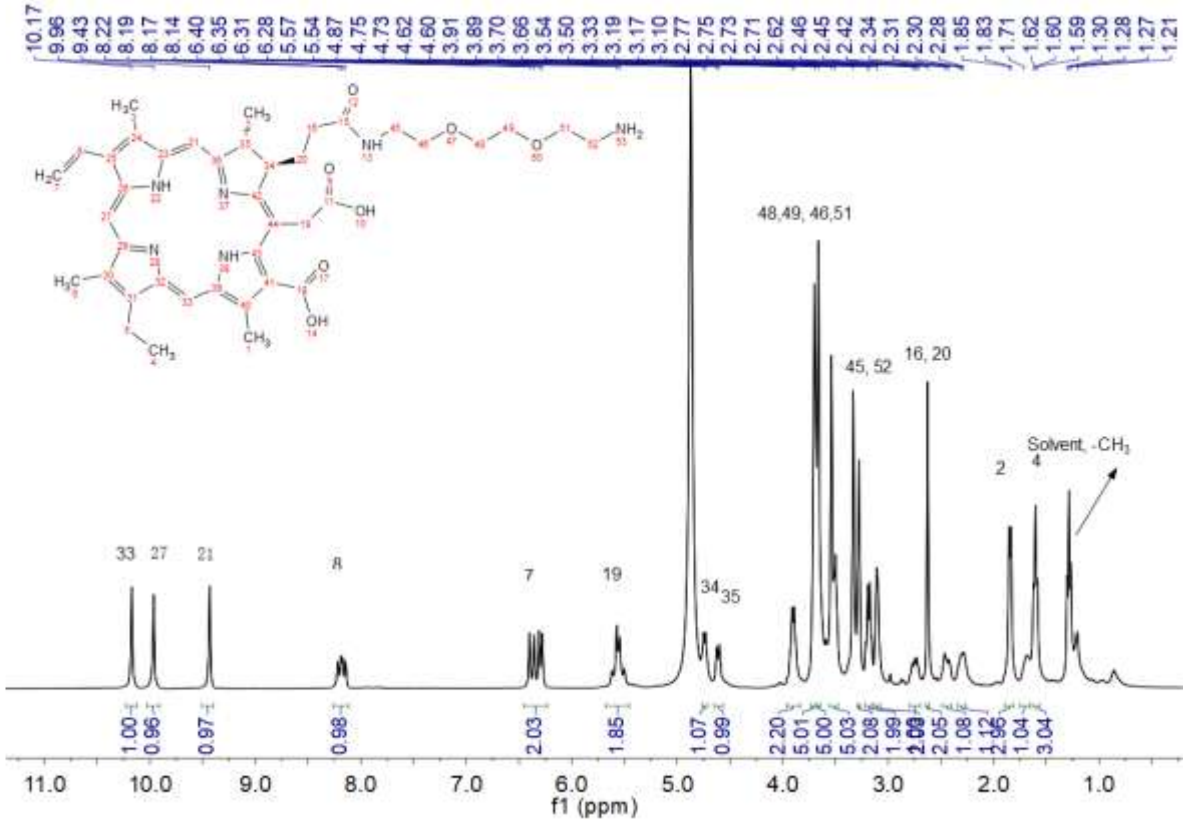

Supplement: Supplementary file 1 — Supplementary Material [file SMSC-2-2100082-s001.pdf]
